# New models of health and social care for people in later life: mapping of innovation in services in two regions of the United Kingdom using a mixed method approach

**DOI:** 10.1186/s12913-024-11274-8

**Published:** 2024-07-15

**Authors:** Helen Frost, Tricia R. Tooman, Navneet Aujla, Bruce Guthrie, Barbara Hanratty, Eileen Kaner, Amy O’Donnell, Margaret E. Ogden, Helen G. Pain, Susan D. Shenkin, Stewart W. Mercer

**Affiliations:** 1 Advanced Care Research Centre, Usher Institute, University of Edinburgh, Usher Building, 9 Little France Road, Edinburgh, EH16 4UX UK; 2 Population Health Sciences Institute, Newcastle University, Newcastle, UK; 3 NIHR Applied Research Collaboration North East and North-Cumbria, Newcastle University, Newcastle, UK

**Keywords:** Innovation, Health and social care reform, Models of care, Older people, Technology, Health inequalities, Evaluation

## Abstract

**Background:**

Innovation for reforming health and social care is high on the policy agenda in the United Kingdom in response to the growing needs of an ageing population. However, information about new innovations of care being implemented is sparse.

**Methods:**

We mapped innovations for people in later life in two regions, North East England and South East Scotland. Data collection included discussions with stakeholders (*n* = 51), semi-structured interviews (*n* = 14) and website searches that focused on technology, evaluation and health inequalities. We analysed qualitative data using framework and thematic analyses. Quantitative data were analysed descriptively.

**Results:**

One hundred eleven innovations were identified across the two regions. Interviewees reported a wide range of technologies that had been rapidly introduced during the COVID-19 pandemic and many remained in use. Digital exclusion of certain groups of older people was an ongoing concern. Innovations fell into two groups; system-level ones that aimed to alleviate systems pressures such as preventing hospital (re)admissions, and patient-level ones which sought to enhance health and wellbeing directly. Interviewees were aware of the importance of health inequalities but lacked data to monitor the impact of innovations on these, and evaluation was challenging due to lack of time, training, and support.

Quantitative findings revealed that two thirds of innovations (*n* = 74, 67%) primarily focused on the system level, whilst a third (*n* = 37, 33%) primarily focused on the patient-level. Overall, over half (*n* = 65, 59%) of innovations involved technologies although relatively few (*n* = 12, 11%) utilised advanced technologies. Very few (*n* = 16, 14%) focused on reducing health inequalities, and only a minority of innovations (*n* = 43, 39%) had undergone evaluation (most of which were conducted by the service providers themselves).

**Conclusions:**

We found a wide range of innovative care services being developed for people in later life, yet alignment with key policy priorities, such as addressing health inequalities, was limited. There was a strong focus on technology, with little consideration for the potential to widen the health inequality gap. The absence of robust evaluation was also a concern as most innovations were implemented without support to monitor effectiveness and/or without plans for sustainability and spread.

**Supplementary Information:**

The online version contains supplementary material available at 10.1186/s12913-024-11274-8.

## Background

Health and social care systems are under ongoing reform in the United Kingdom (UK) in the context of an ageing population and increasing cost pressures from a programme of austerity introduced in 2010 [1–5]. Health and social care reform is a top priority for governments in England and Scotland [6], with both countries having policy priorities on preventative care, mental health services, person-centred care, the use of new technologies, and reducing or mitigating health inequalities by providing equity of access to high quality care or targeting specific interventions in deprived areas [6]. However, despite these major policy drives, the extent of ground-level innovation to meet the needs of all older people is unclear [7, 8].

Multimorbidity is defined as the occurrence of two or more health conditions. It is more common at older ages, and occurs earlier for people living in socioeconomically deprived areas [4, 9]. Healthcare systems and national clinical guidelines, however, remain largely organised around a single-disease model [10]. When applied to people with multiple conditions, a single-disease approach can lead to fragmented care, poor patient experience, futile treatments with risk of harm and inefficiencies that increase system costs [11]. Recent synthesis of research evidence on new models of care delivery identified 66 reviews, involving 1272 primary studies [12]. All 1272 primary studies took a single-disease or single condition approach. There is thus an urgent need for innovations and new models of care that better meet the needs of older and disadvantaged people, involving health and care systems that collaborate to address multimorbidity.

Service provider organisations from health, social care and the non-statutory sector are continuously innovating to respond to new challenges or opportunities. One example is the Person centred coordinated care (or P3C) work based in South West England, UK. These efforts have involved bringing together primary, secondary, community and voluntary sectors with embedded researchers in order to codesign new models of care to address, amongst other challenges, system fragmentation and depersonalised care that results in preventable hospital admissions [13–15]. However, in most places information on ground level innovation is difficult to access – they are seldom reported in academic journals and there is currently no source of collated data. Our understanding of the extent to which policy priorities are being addressed [16, 17], or where innovations in models of care are targeted is incomplete.

## Methods

### Aim, design and setting

The aim of this study was to identify and map recent innovations in new models of care that support the health and/or social care of people in later life in South East (SE) Scotland and North East (NE) England, with a particular focus on understanding the role of technology, whether or not innovations were formally evaluated, and whether innovations addressed health inequalities.

A mixed-methods approach was used to identify and explore innovations in health and social care across NE England and SE Scotland:

1. Discussions with key stakeholders in health and social care identified through contacts in existing networks.
2. Semi-structured interviews with stakeholders identified through the above method to explore innovations in greater depth.
3. Searches of health and social care related websites from April 2021 to December 2023 for relevant information.

### Selection criteria for mapping innovations

Innovation has been defined as, “health practices, systems, products and technologies, services, and delivery methods that result in improved healthcare” (p89) [18]. For this mapping exercise, we defined innovation as a new initiative, or one built on previous or existing work, that focused on a new way of delivering, or facilitating the delivery of, health or social care [18, 19]. We were interested in innovations that were:

- Focused on later life, which we defined as older people aged 65 years + for the general population and 50 years + for those living in disadvantaged areas [9]. This definition also included people referred to as elderly, older adults, older persons, and older people or seniors living in their own homes or in health and care settings [20].
- Based in NE England or SE Scotland. In NE England we followed the footprint of the Integrated Care System for North East and North Cumbria [21], which has a population of 3.1 million people, of which 32% live in the lowest quintile of deprived areas. In Scotland we looked across the South-East Health and Social Care Region, population 1.3 million, with 12.3% in the lowest quintile of deprived areas (Scottish Borders 6.3%; Fife 19.8%; Lothian 10.9%).
- Focused on multimorbidity or frailty. Innovations that focused on care for single diseases only (e.g., dementia) were therefore excluded.
- Involved older people, and were of particular relevance to older people. However, we did not exclude innovations that could be universal for all age groups (e.g., green social prescribing).

### Stakeholder sampling and recruitment

We identified key stakeholders by first drawing on the research team’s networks, followed by website searches. Our multidisciplinary team was comprised of clinical and academic experts in health and social care, including primary care, geriatric medicine, psychology, health services systems improvement, public health, and two experts by lived experience (Patient and Public Involvement and Engagement (PPIE) members).

A purposeful sampling approach and snowballing strategy were adopted [22] and involved all team members. The sampling aimed to include geographical, multi-professional and multi-sectorial (e.g., statutory bodies, third sector) representation of stakeholders for people in later life in the two regions and innovations in different physical settings across the care context, i.e., primary care, care homes, other community-based settings. Two researchers had 51 exploratory discussions with stakeholders and conducted 14 semi-structured interviews to explore the innovations in more depth (see Table 1). A general job description, sector and region of the innovation service providers interviewed is shown in Table 1.

**Table 1:** Description of interviewees

Sector	Job Description	Region
H&SC	Program Lead	NE England
Third sector	Service Manager	NE England
Third sector	Innovation Director	NE England
Social care	Service Lead 1	NE England
Social care	Service Lead 2	NE England
H&SC	Service Lead	NE England
Third sector	Program Lead	NE England
Social care	Academic	SE Scotland
H&SC	Service Lead	SE Scotland
Health care	Service Manager 1	SE Scotland
Cross sector	Technology Lead	SE Scotland
Health care	Service Manager 2	SE Scotland
H&SC	Service Manager	SE Scotland
H&SC	Strategic Program Manager	SE Scotland

Informed consent was obtained from individuals prior to conducting interviews and were carried out between September 2021 and December 2021. The project was reviewed by the Research Ethics Committee at the University of Edinburgh (21-EMREC-025) and was deemed to be a service evaluation.

### Data collection for mapping work

For each innovation, we recorded the following data (see Table 2), broadly developed from the ‘Ten steps to making evaluation matter’ Framework [23] and for technological innovations the ‘Non-adoption, Abandonment, Scale-up, Spread, and Sustainability’ (NASSS) Framework [24]. These frameworks informed our mapping inquiry as we were interested in a range of innovations, including technology-based ones.

**Table 2:** Data collection categories

• What is the overall aim or vision of the innovation? • Who designed and funded the innovation? • Who are the target population and what is the setting/care context? • What are the components of the innovation? • Is there an underpinning evidence-base? • Is technology included? • Is there an intentional focus on health inequalities? • Has the innovation been implemented? If so, how, where and by whom? • Has the innovation been evaluated? If so, how, where and by whom? • Is there any prior evaluation? • Does the innovation have potential to be tested in a Randomised Controlled Trial (RCT) or natural experiment? • Is there potential for scalability and generalisability?	

Source material drawn from: ‘Ten steps to making evaluation matter’ Framework [23] and ‘Non-adoption, Abandonment, Scale-up, Spread, and Sustainability’ (NASSS) Framework [24]

### Data collection for the interviews

The topic guide for interviews (see Appendix 1) was also informed by the ‘Ten Steps’ Framework [23] and, where appropriate, the NASSS Framework [24], and was iteratively developed in consultation with the project team including our PPIE members. The topic guide was piloted in two interviews. Interviews were conducted by two researchers online (via MS Teams) or by telephone and lasted from 30 to 52 min (average of 49 min). Encrypted recording equipment was used to capture participants’ oral consent and, separately, interview data. Pseudonymised audio files were then stored on the University of Edinburgh’s DataStore, accessible only to the two interviewers. Recordings were uploaded to a transcription service scrutinised and approved by the University of Edinburgh to meet strict data protection requirements. Interviewers subsequently removed all identifiable information in the transcripts before analysis was conducted involving additional members of the research team.

### Data analysis

The research team reviewed and agreed an initial coding framework based on the theory-drawn categories developed for the mapping work (see Table 2) and summarised each innovation in tables for NE England and SE Scotland (see Supplementary files). Exploratory discussions with stakeholders added depth to our understanding of innovations. Interview transcripts were coded deductively using the framework-based categories and analysed using the framework approach [25]***.*** Innovations that included any type of technology were identified (e.g., communication platforms, sensors, digital tools for monitoring and/or predicting events such as falls).

Each innovation was reviewed to determine whether formal methods for evaluation had been used at the innovation site, and whether stakeholders or innovation websites stated that health inequalities were considered. SPSS Version 28.0.1.1 was used for the descriptive analysis. Finally, data summary and framework tables were imported to NVivo 12 qualitative data analysis software and were analysed thematically to identify further patterns within the full set of innovations [26].

Our mixed methods analysis used an explanatory sequential design [27] where the quantitative data collection and analysis was supplemented by the qualitative interview data. The quantitative data analysis involved all sampled innovations, which provided an understanding of the whole, whilst the qualitative data complemented this broad understanding by providing a nuanced understanding of specific innovations. Together these data enabled both a wide lens and specificity for understanding innovations in these two regions. Furthermore, regular team meetings involving broad expertise and perspectives, including especially PPIE members, provided the opportunity for discussion and reflexive checks on our interpretation of these data [28].

## Results

We identified 111 innovations of new models of care (57 in NE England and 54 in SE Scotland). Eight of the innovations were still in the planning stage, and the rest were either already implemented or in the process of implementation. We begin by describing our qualitative findings, specifically in relation to technologies, health inequalities and evaluations, and then examine the focus of change for the innovations, whether they were at the system-level or patient-level. Finally, we put these into a broader descriptive context based on our quantitative findings.

### Qualitative findings

#### Technologies

We found both a range of technologies used, and range of complexity involved in the technologies deployed. On one end, there were innovations involving well-established forms of technology such digital platforms that consolidated information for online access and facilitated communication. Examples included the provision of online consultation and therapy as well as online movement groups set up during the Covid-19 pandemic to support older people to remain active. Technology was also a key component of more complex models of care, for example, specially configured homes equipped with a range of technologies to support frail residents. More advanced technologies (which were less common) varied and ranged from the use of robots to support older people in the community, to bedside sensors to help prevent falls, and predictive tools to detect frailty or functional decline. Data collected via technology was also used in innovative ways – for example, employing artificial intelligence to provide personalised advice for older adults with long-term conditions. Other innovations used technology to align and integrate data collection for greater efficiency. For example, a wound care service for older people living in the community or care homes allowed care home and nursing staff to upload photos that automatically integrated with the Electronic Patient Record. Some technologies were viewed by clinicians working across acute and community care as a highly valuable addition to support service delivery. For example,

*The* [named digital] *documentation system has been huge for us… previously* [software] *only captured attendances, just the general attendances…whereas* [name] *actually gives us data on the number of occupational therapy sessions, what’s done, the outcomes, physiotherapy, the nursing sessions. So, it captures things in a lot more detail within the team, rather than just what’s happening to the service.* (Health Care Service Manager 2, SE Scotland)

Other providers were planning to further develop their use of technology to support service delivery in the future:

*We’re trying to think about things like in ten years’ time the people who will be going in to needing services, older people's services, are probably in their 60s now, so they’ll be probably 70s or 80s. And with their, all of the embraced technology that's had to happen through Covid we're going to have people who are a bit more tech savvy than we were expecting.* (Social Care Service Lead 1, NE England)

However, interview participants also acknowledged a range of challenges in using technology in services for older people*.* For example:

*The technology is all there. We just have to understand how to leverage these existing technologies in an ethical way…Issues are equality, equal access, issues are accessibility of these technologies, issues are ethics, issues are the cost, issues are the ignorance. So, it’s a matter of the education of the people.* (Third Sector Innovation Director, NE England)

Further challenges included having the appropriate infrastructure in which to embed technologies, and the ethics and costs involved in using technologies in healthcare, which impacts on those vulnerable to digital exclusion.

#### Health inequalities

When prompted, interviewees were aware of the importance of health inequalities and related policy directives.

*There’s a new fund that’s come through from the government on community mental health and wellbeing. And it’s got a real focus on inequalities. So, we’re using it as an example of community commissioning…I think that will give us some learning for how we do that wider across the city and how do we do stuff that is proportionate.* (H&SC Strategic Program Manager, SE Scotland)

Most interviewees did not have any data on how their innovative work helped disadvantaged people or whether it widened the care gap:

*In terms of other aspects of equality, then I guess it’s hard for us to know what we’re missing but we still wish to explore it, people who are homeless or people in very deprived and socioeconomic situations. So, it’s that side of things that we’re probably not reaching, although we don’t know the extent of that yet but we’re very aware that we could be missing people.* (Health Care Service Manager 2, SE Scotland)

*Well, we haven’t directly* [considered health inequalities]*, I guess. But again, probably because of the stage it’s at. It’s on our radar, but I don’t think at this point we have sort of thought*. (H&SC Service Lead, SE Scotland)

Of the innovations that sought to address health inequalities, four developed and delivered social prescribing-based work. Three innovations embedded navigators/link workers in primary care settings to signpost patients to community-based activities and support. Social prescribing is an alternative to pharmacological treatment that has potential to reduce GP workload [29, 30]. It addresses issues that influence health status and helps patients take greater agency over their health and wellbeing. Another innovation was a wide collaboration using systems-thinking to harness the complex benefits of urban green and blue spaces for maximising health and wellbeing, with a focus on reducing health inequalities [31, 32]. One innovation used artificial intelligence to detect and assess levels of pain for those unable to verbalise their discomfort and another introduced a digital surgery, which they said helped address inequalities by providing patients choice around how to interact with their GP. The remaining innovations that noted health inequalities as part of their remit included the provision of social activities such as Men’s Sheds and dance classes for older people, as well as telehealth and domiciliary care.

#### Evaluation

Various methods were used to evaluate the new ways of working. Some innovations were part of a wider initiative that involved evaluation. For example, two were introduced under the NHS England Vanguard program, and Age UK led a Personalised Integrated Care Programme [33] involving multiple sites. The latter integrated health, social and voluntary sectors to provide personalised care for older people, with the aim of reducing unanticipated hospital admissions and associated costs (which it did not achieve) [34].

Few evaluations included any economic evaluation. Those that did, found it challenging to demonstrate cost savings. For example, a falls rapid response service noted that it was difficult to track financial savings because the primary benefit of the service was realised when people were *not* admitted to hospital. Measures of activity, such as how many patients were seen, was unhelpful in estimating savings. Some evaluations incorporated acceptability of the innovation by patients, staff, or both. “*What they’re doing is, you know, do the staff like it, do they find it useful, do they use it, you know, so it’s more about usability, acceptability, that they’re doing.”* (Cross Sector Technology Lead, SE Scotland).

Most service providers understood the importance of evaluation but faced time limitations, pressures to deliver rather than evaluate care, and a shortage of funds to support it.

*We need time to evaluate and write up bare minimum...ideally an evaluation report. So, we did that for the Near Me projects. We didn’t ask too much of care homes in terms of the data they gave us for that one because we recognised how much they were struggling.* (H&SC Service Manager, SE Scotland)

As a result, assessments were either limited or, indeed, not done at all (as noted above, 68 or 61%): *“In terms of evaluation, we don’t have any form of evaluation planned.”* (H&SC Program Lead, NE England)

#### Focus of change for identified innovations

As ‘innovating’ involves change, we examined the focus or level of change innovations targeted, specifically who was ‘being innovated’ or at what level was change involved. On this basis, we were able to group them based on whether change was primarily at the health and/or social care *system-level* or primarily at the *individual patient-level*. We found that system-level innovations sought to change professional and organisational behaviour and were primarily aimed at reducing system pressures and increasing system efficiencies. Examples included efforts to prevent escalation of care, with a common goal to reduce hospital (re)admissions (see Table 3). By comparison, patient-level innovations focused directly on individuals and sought to enhance patient health and wellbeing. These innovations involved supporting older people to remain independent longer, including ways to better self-manage their conditions and the provision of activities that incorporated mental, physical and social support (see Table 4). We explore these two categories below in our quantitative data findings.

**Table 3:** Examples of systems innovations to reduce system pressures

*Example 1—Preventing escalation* **Wellbeing Independence Service, South Tyneside Council** Older clients discharged from hospital who, following assessment, still have care needs. To help get people home, improve their independence and prevent readmission to hospital provides 6-weeks of support personalised to the client’s needs, with a further 6-weeks of support provided if needed. Includes an Alcove, a two-way communication device, to enable communication with the client up to 4 times a day and helps connect them to family. Daily contact if the client has not answered that day. Also includes an automated pill dispenser, ‘Your meds’ to monitor tablet-taking each day. Clients receive a 15-min call at the end of each day to prompt them to take their tablets for that day if they have not already. *Example 2—Increasing efficiencies* (e.g., to reduce pressures on primary care) **BP@Home, Health Call in partnership with Teams Medical Practice** Links people living at home who are required to check their blood pressure over a specific period with general practice. Supports remote monitoring of blood pressure and improves current and future self-management, saves time for patients and clinicians, reduces costs for GP phone calls and patient travel to and from the GP practice for multiple appointments, and improves clinical outcomes through early detection of accelerated hypertension. Features include patient communication using text message or email for advice, e.g., a tutorial video for taking blood pressure at home, patient/clinician alerts for potentially life threating readings, and clinician alerts for non-responders and end of 7-day monitoring alerts. Blood pressure average is automatically calculated with an option to output a PDF document of readings to the patient’s record. SNOMED code is mapped back into patient’s clinical record and integrates with EMIS Web and System One. *Example 3—Offering an advanced care practitioner response* **Advanced practitioner home visiting service, North East Ambulance Service** For older people in the community with complex health needs Uses advanced practitioners to carry out home visits of vulnerable patients and reduce pressure on general practice and unnecessary hospital admissions. Paramedics are upskilled to advanced practitioners to provide more generalist care of the complexity seen in general practice, including falls, long-term conditions, mental health issues in older adults (65 +).

**Table 4:** Examples of individual innovations to improve health and wellbeing of patients

*Example 1 – Improve physical health* **Make Movement your Mission (MMYM) providing activities to improve physical and social health (online)** Make Movement your Mission (MMYM) set up during the COVID-19 pandemic with an overall aim to promote physical activity levels in older people and promote movement into daily activities, whilst at home. The tutors, run the sessions three times a day aiming to get the circulation moving, mobilising joints and muscles to increase functional strength and balance moves and balance exercises. *Example 2 – Improve well-being of patients at end of life and support care home staff* **Online supported Conversations and Reflections in relation to death and dying in care homes (OSCaRS)** To support and train care home staff in conversations and reflections in relation to death and dying in care. The innovation includes online emotional support and practice-based learning on death/dying and end of life care. Research suggests the OSCaRS are: 1) feasible and acceptable to carry out 2) inexpensive 3) valued by the care home workforce Further work is ongoing to develop online support and resources in relation to death/dying including education, training and staff wellbeing resources and implementation *Example 3 – Enabling better self-management* **House of Care** Supported by ‘The Alliance’ in collaboration with the six partnership areas across Scotland including (Lothian/ Thistle Foundation and RCGP) For people living with multiple long-term conditions across all settings. Enables and supports conversations about care and support planning for self -management. Involves a tool that focuses on shared decision making, partnerships, and conversations about what matter to people, engagement, and empowering people to self-manage enabled by informal and formal sources of support and organisational/policy support.	

### Quantitative findings

Of the 111 innovations identified, 74 (67%) focused primarily on change at the system-level and 37 (33%) primarily at the patient-level (Table 5).

**Table 5:** Characteristics of identified innovations overall and at the system level and patient level

	**All data (*n* = 111)**	**Systems focus (*n* = 74)**	**Individual focus (*n* = 37)**
***Sectors of identified innovations***			
Health care	38 (34%)	35 (47%)	3 (8%)
Social care	14 (12%)	5 (7%)	9 (24%)
Health and social care	23 (21%)	18 (24%)	5 (13%)
Third sector	12 (11%)	1 (1%)	11 (30%)
Industry	11 (10%)	3 (4%)	8 (22%)
Cross-sector	13 (12%)	12 (16%)	1 (3%)
***Settings of identified innovations***			
Primary care	13 (12%)	11 (15%)	2 (5%)
Care homes	19 (17%)	16 (22%)	3 (8%)
Inpatient hospital care	7 (6%)	7 (9%)	0 (0%)
Community based	45 (41%)	20 (27%)	25 (68%)
Multiple settings	27 (24%)	20 (27%)	7 (19%)
***Included technology***	65 (59%)	42 (57%)	23 (62%)
***Types of technology***			
Information	8 (7%)	4 (5%)	4 (11%)
Communication	18 (16%)	12 (16%)	6 (16%)
Tools	15 (14%)	14 (19%)	1 (3%)
Data Integration	12 (11%)	10 (14%)	2 (5%)
Advanced	12 (11%)	2 (3%)	10 (27%)
None	46 (41%)	32 (43%)	14 (38%)
***Included evaluation at innovation site***	43 (39%)	35 (48%)	8 (22%)
***Methods of evaluation used***			
Quantitative	12 (11%)	10 (14%)	2 (5.4%)
Qualitative	8 (7%)	6 (8%)	2 (5.4%)
Mixed/multi methods	18 (16%)	16 (22%)	2 (5.4%)
Unclear	3 (3%)	1 (1%)	2 (5.4%)
In development	2 (2%)	2 (3%)	0 (0%)
None	68 (61%)	39 (52%)	29 (78.4%)
***Innovations in development***	8 (7%)	5 (7%)	3 (8%)
***Focused on health inequality***	16 (14%)	8 (11%)	8 (22%)

#### Sectors leading identified innovations

Service providers from a range of sectors were involved in new ways of delivering care to people in later life. Within these innovations, the health care sector alone was the most common, with integrated health and social care innovations being the next largest sector. However, this varied between the system-level and patient-level innovations, with the latter having most innovations led by the non-statutory or third sector (Table 5).

#### Settings of identified innovations

Innovations spanned several settings. Overall, the commonest setting overall being community-based provision of care followed by innovations that took place across multiple settings. Many of the patient-level interventions were in the community setting (Table 5).

#### Included technologies

Over half of all innovations involved technology of some kind (59%) (Table 5). Technology innovations were placed into five categories, based on the function they predominantly served. *Information* technologies provided online access to information. *Communication* technologies enabled digitally based communication and two-way sharing of information, for example the telehealth innovations. *Tools* involved digital mechanisms that produced useable outputs, and for these innovations involved mainly screening and prediction tools. *Data integration* technologies focused on the consolidation of different information systems for efficiency, such as integrating records and identifying available capacity. Finally, *Advanced* technologies were those that involved assistive devices, including robotics, sensors, wearables, or incorporated artificial intelligence.

**Fig. 1:**
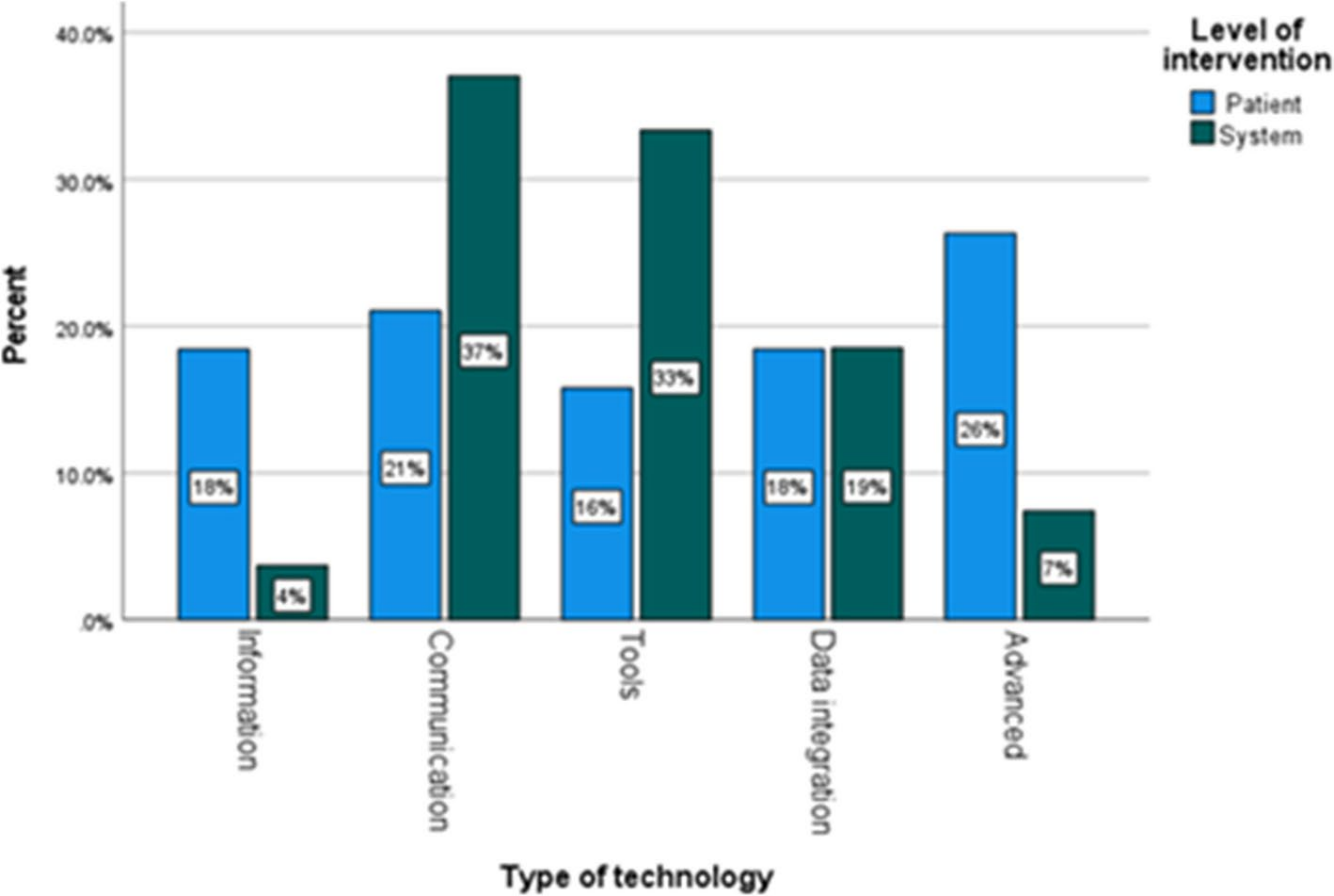
Distribution of technologies used in patient-level and system-level innovations

Technologies that enabled communication were the most common form of technological innovation overall, with Tools being the second most common (Table 5). However, at system-level, the commonest technology was Tools, whereas at the patient-level it was Advanced Technologies (Table 5). The distribution of types of technology used (for those interventions that used technologies) at the two levels is shown in Fig. 1.

#### Health inequalities

Only 16 (14%) of all innovations had a focus on health inequalities, and four of these were still in the planning stage. Innovations focusing on health inequalities were twice as common at the patient-level than at the system-level, although were still rare (Table 5).

#### Evaluation

Evaluation was identified as completed or planned/in progress in a minority of innovations (39%), and was twice as common in the system-level innovations than in the patient-level ones (Table 5). Mixed methods were most often used in evaluations, followed by quantitative approaches (Table 5), which included surveys, observational studies, and case control studies. There were no randomised controlled trials. At the system-level, mixed-methods and quantitative methods were the most common evaluation approaches, whereas the small number of evaluations at patient-level were equally distributed across the different approaches (Table 5).

## Discussion

### Summary of findings

This study mapped innovations that supported the health and social care of people in later life in two regions of the UK. We identified 111 innovations relating to new models of care, many of which had been rapidly developed during the Covid-19 pandemic. They fell into two broad groups: (1) innovations to address system pressures (e.g., preventing unplanned hospital admissions); and (2) individual-level innovations to enhance health and wellbeing and provide support for independent living. From these data we see that when the health care sector is involved, change is predominantly focused on making changes to the system, whereas for social care and the third sector, efforts concentrate directly on helping individuals change. The latter is perhaps unsurprising as the key role of the third sector is helping communities rather than statutory services, and thus one would expect more third sector innovation at the patient level change. Nearly half (41%) of innovations took place in community settings. This aligns with governmental policy directives to shift care away from (costly) institutional settings into community settings that provide new models of care and enable people to live at home or in a homely setting. As older populations continue to expand, reforms that aim to enable more efficient systems operations and keep older people in their homes are becoming increasingly common. We discuss many of the implications of our findings below, but a key implication for older people themselves is the fragmentation of care than can result from numerous innovations taking place in different settings and sectors. Fragmentation of care is a key concern for people with complex needs, such as multimorbidity or frailty [35] and further rapid expansion of innovations by different players (industry, third sector, healthcare, social care) is likely to fragment care further. There is increasing evidence of the importance of continuity of care [36] yet in the systems currently in operation in Scotland and England, the role of ‘care-coordinator’ is often left to individuals and families [37].

Almost two thirds of innovations involved technologies of some form, but the use of advanced technologies was uncommon overall (though more common in patient-level innovations). Although four out of ten innovations had a formal evaluation of some type, but robust and/or external evaluation were uncommon. Qualitative findings highlighted both benefits and challenges related to the innovations, their implementation and evaluation. Health inequalities had not been a major consideration in implementing change, with fewer than one in seven innovations having this as a primary focus. Digital exclusion of certain groups of older people was an ongoing concern. For staff, challenges lay in finding the time to conduct internal evaluation and the lack of research skills and training.

### Relationship to literature

Health and social care policy across England and Scotland has a growing narrative around integration within healthcare and across health and social care boundaries [38, 39], adult social care reform [40, 41] and the importance of reducing health inequalities [6]. The findings of this study echo a previous review that looked at more than a decade of national pilot programs in England. That study identified barriers to better coordination of services, including short timescales, poor professional engagement, information and data sharing problems, and conflicts with changing national policy [42, 43]. The definition of evaluation that we adopted was broad and included any formal study design, such as qualitative study, pilot study, case study, or audit/health improvement project, RCT, or mixed-methods approach. It is noteworthy that in our study, fewer than half of the innovations had any form of evaluation, but this is still higher than in the previous evaluation of new models of care in Scotland [32]. 

Technology was incorporated into many of the identified innovations. Digital approaches were introduced rapidly during the Covid-19 pandemic and although of potential benefit to those who could use the technology, the pandemic may have widened digital inequalities for many, including older people [44–46]. Our findings described barriers to using data (e.g., data sharing) and implementing digital technology, despite a policy drive to increase use of technology in healthcare [47, 48]. Furthermore, the increased emphasis on technology in innovations both on the systems and individual levels is predicated on an assumption that digital access and literacy are equitably dispersed. However, for all the benefits of providing information and moving communications into online platforms are for some, for others this further exacerbates health inequalities [49].

An evaluation of over 200 new models of care between 2016–2018 in Scottish primary and intermediate care reported similar findings on health inequalities. Only one in 10 was focused on reducing inequalities, despite that being a stated aim of the programme [50]. The lack of focus on inequalities clearly chimes with the findings of the present study, despite the importance of addressing health inequalities being a key thread in several policies across both countries (e.g., [51, 52]). The King’s Fund has reported that lack of innovation for people from disadvantaged areas is an ongoing challenge [53]. Their report calls for action at all levels of the health and care system is required [53]. For example, while social prescribing innovations provide promising alternatives to pharmacological interventions, there are concerns that the inverse care law is once again at work [54]. Those with ready access to a variety of activities benefit, whilst those who live in more deprived areas do not [55]. Where social prescribing has been shown to be effective, people need to feel they have similar interests and a commonality of life experiences [56]. Furthermore, the individual partaking in social prescribing activities must have the capacity to try a new endeavour. For some of the most vulnerable, this may not be the possible [56]. Innovations may have an unequal impact on different socioeconomic groups that can inadvertently generate or widen inequalities for individuals [57]. 

### Strengths and limitations

A strength of our work is that we have provided a systematic snapshot of innovation of what is happening on the ground for older people across two major regions in the UK. There are few reports of this kind in the published literature. Without this information, improvements and gaps in services remain poorly understood, and as a result are unclear how to move an innovation forward and closer to full-scale implementation or a new model of care.

We acknowledge the paucity of guidance on how to carry out mapping work of this kind. Our approach was similar to ‘horizon scanning’ [58] that helped us capture a range of innovations across the spectrum of care for older people. However, a snowball approach is limited to local and regional contacts identified through websites and our own existing networks, and this strategy misses relevant work outside these networks. Additionally, with grey literature often difficult to locate, conducting data collection of this type in a rigorous manner was challenging. We accepted it would be impossible to capture everything and thus our list of innovations is not exhaustive. Furthermore, we excluded disease specific innovations that at times overlap with multimorbidity, e.g., dementia. Finally, this work did not map the views and experiences of those receiving the innovations. We recognise that understanding service users’ experiences, including those of informal carers, is important. We worked with patient and public partners throughout the design and implementation of this research, nonetheless, more could be done to capture service users’ experience in future research [59, 60].

### Implications for policy, research, and practice

The current project highlighted the need for evaluation of innovations as they emerge and to avoid ‘pilotitis,’ which is characterised by numerous small pilot studies, funded for short periods of time, and with little or no pre-planned evaluation [8]. Without evidence to demonstrate impact, and in particular impact that meets the stated needs of people receiving care [61], innovations are unlikely to be sustained even when they are helpful. Previous attempts to map innovations in care for people in later life in Scotland, reported over a decade ago, recommended more rigorous evaluation of newly developed innovations, yet our work suggests robust evaluation remains sparse [62].

We have previously suggested a need for ‘middle ground research’ involving NHS–academic collaboration to co-create and rigorously evaluate innovations and new models of care as a highly productive way to develop evidence of effectiveness, to facilitate translation into widespread practice, and to ensure the evaluation of real-world implementation [63]. A commitment to ‘middle-ground research’ and co-production with those using the services, as seen in the Person centred coordinated care work [13–15] that included evaluation expertise, can help facilitate robust evaluation even of small-scale innovations and models of care, and build in thinking and develop expertise for future evaluations of effectiveness and cost-effectiveness of large-scale trials and wide-spread implementation from the start.

The added value of having external support for evaluation, for example through the National Institute for Health and Care Research (NIHR) Applied Research Collaboration (ARC) program across England [64], is also clear, and such collaboration is to be encouraged.

We identified a limited focus on addressing health inequalities in innovations, which is of concern given that inequalities are widening in both England and Scotland [44, 45]. From our findings and the wider literature, we would argue that innovations should always include consideration of the potential for social patterning of their impact, and that this should be monitored. However, health inequalities are exacerbated by the ‘ongoing Inverse Care Law’ in the UK, in which access to high-quality health and social care is worse for disadvantaged communities with the poorest health [4, 65]. This is further compounded by the widespread shortages of general practitioners in many areas of the UK. Thus a ‘systems thinking’ approach is required when implementing new models of care, so that consideration is given to the possible wider impacts on innovations within health and social care systems [48].

We would argue that people in later life themselves should be involved in the development of new models of care, as recommended in the MRC Framework for developing and evaluating complex interventions [66]. More work is needed around co-production with older people to develop innovations that are designed for them to ensure that they address their needs, and the approaches are feasible and acceptable to them [67–71].

## Conclusions

This mapping exercise demonstrated that the response to policy calls for innovative reform has resulted in a wide range innovative care services being developed for people in later life across NE England and SE Scotland. There was a strong focus on technological innovation, however, some caution is needed to ensure that a growing focus on technology does not further generate health inequalities, given the risk of digital exclusion for older people and people from deprived areas. Health inequalities received scandalously little attention. Despite consistent calls to address ever widening health and care inequalities, few innovations appear to do so and for those with intentions to address health inequalities, there was little evidence of how health inequalities would be monitored. Furthermore, services were under resourced, and most health and social care providers lacked capacity and training in evaluation. The result being that the evidence base for new models of care remains weak. Without robust evaluation, resources consumed by implementing unproven innovations risk being wasted and worse, may widen the health inequality gap.

### Supplementary Information

Supplementary Material 1.

## Data Availability

Most of the data supporting the findings of this project are provided in the Appendix in the Supplementary Files. The rest is available from the corresponding author, SWM, upon reasonable request.

## Abbreviations

NASSS: The ‘Non-adoption, Abandonment, Scale-up, Spread, and Sustainability’ (NASSS) Framework
NEE: North East England
NHS: National Health Service
PPIE: Patient and Public Involvement and Engagement
SES: South East Scotland
UK: United Kingdom
