# Supplementary material for: New models of health and social care for people in later life: mapping of innovation in services in two regions of the United Kingdom using a mixed method approach
Source: BMC Health Serv Res. 2024 Jul 15;24:812. doi: 10.1186/s12913-024-11274-8 (PMC11247813; doi:10.1186/s12913-024-11274-8)
Supplement: Supplementary file 1 — Supplementary Material 1. [file 12913_2024_11274_MOESM1_ESM.docx]

**Topic guide used in semi-structured interviews with key stakeholders**

|  | Suggested Questions (*Prompts in italics)*  The term ‘innovation’ is used throughout but could be project / policy / programme / innovation /intervention as appropriate, depending on stakeholder interview |
| --- | --- |
| Introduction | **Tell me about your role in later life care.**  **-***How long have you been working in this position?*  *-What is your previous experience?*  **1.** **Which population(s) and setting (s) do you focus on?**   - *Can you tell me a little more about the setting you work in (size, demographics, patient groups)?*   **2. To what extent does your work cover the following four transitional points:**   - Primary care, community, and third-sector support for people in later life living in the community. - Integration of statutory health and social care services for people in later life living in their own homes. - Transitions into care homes. - Improving the care of those already living in care homes. |
| Programme planning, Theory, Evidence, and anticipated Impacts | **3.** **Tell me about the specific problem associated with ageing for which this innovation/s was [is being] designed.**  *Prompts:*  *-Would people need input from more than one specialist or be in more than one care pathway?*  *-Do some of the people affected have cognitive impairment, learning difficulties or communication difficulties e.g. hearing ?*  *-Do people with the condition tend to be in multiple care pathways or see more than one provider? E.g. link workers, GP AHP.*  **4**. **Tell me more about the innovation (s) in later life care that you are aware of in your field of work**  ** Focus on specific innovations mentioned.  **5.** **What’s your role in the delivery of this service?**  *Prompts:*  -*Where does your professional role (or remit) begin and end for this patient group?*  *-To what extent do you believe that current pathways of care meet patient needs?*  **6**. **Tell me about the overall aim or vision of the innovation?**  *Prompts:*  *- Is it micro, miso, macro level?*  *-How did it come about?*  *-Who developed the innovation?*  *-Who funds the innovation?*  *-Has anything been written about the development of the innovation?*  *-Has a pilot study been carried out – is it written up? Where and when?*  **7. Tell me more about the different components of the innovation?**  *Prompts:*  *- Are some components more important than others?*  *- What are the main ‘active’ components of the innovation?*  *- To what extent has it been embedded into routine practice?*  *- What is the timeframe for delivery?*  *- Is the current version of the innovation the definitive version?*  *-Have refinements been made?*  ***8. Who works on the innovation?***  *Prompts:*  *- Who are the key care providers of the innovation?*  *- How does it operate in practice?*  **9. What is the underpinning evidence-base? How did the innovation come about?**  *Prompts:*  *- Is the innovation based on theory? What theory (theories) is it based on?*  *- How were users involved in shaping the development of the programme?*  *- How has user involvement changed the intervention, either in the development or subsequently?*  **10.** **To what extent are health inequalities considered in the innovation?**  **11. Do the target population for this innovation have social, cultural, or spiritual factors that need to be considered?**  *Prompts:*  - *Are some of them likely to be socio-economically disadvantaged, homeless, or socially excluded?*  *- Might some have religious restrictions or expectations that would affect how they manage their condition and their acceptance?*  *- Are some likely to have low health literacy (poor understanding of what is wrong and how to manage it)?*  *- Are some likely to have low system literacy (poor understanding of how to navigate the health or care system)?*  *- Are some likely to have low digital literacy (poor understanding of technologies and how to use them)?*  *- Are some of them likely to have problems understanding the language used by staff?*  **12. What is the anticipated impact of the innovation and what is the expected time frame?**  *- Patient/ participant feedback*  *- what are the key outcomes?* |
| Technology questions (*use NASS guide for Technology innovations)* | **Is there any technology involved?**  **If not - Do you think technology in any form could help deliver the intervention / innovation?**  *Prompt:*  *-What might that be and why do you think it could help?* |
| Learning framework and Pathways of Influence | **1. Which policies were involved in the development of this innovation?**  *Prompts:*  *-What were the policy aspirations?*  *-What was the underlying theory?*  *-Are the innovation goals consistent with the policy aspirations?*  *-Is the implementation in alignment with the policy aspirations?*  **2. What organisational structures and processes are needed to support the innovation?**  *Prompts:*  *-Is this funded in the long term?*  *- Has there been any training related to the planning and implementation of the innovation?*  *- Has any time been embedded for personal development?*  **3. What are the barriers/facilitators, or key challenges/opportunities that you faced during the planning and implementation of the innovation?**  *Prompts:*  *What do the frontline staff think of the innovation?*  *Are there any funding issues?* |
| Evaluation | **1. Tell me about evaluation of the innovation.**  *Prompts:*  *- Do you collect any data for audit / evaluation purposes?*  *-if yes - How, where and by whom?*  *- What were the findings of the evaluation?*  *- What did stakeholders and service users think?*  *- How are these data used?*  *- Are data linked to other data and where, how?*  **2. What do patients and carers think of the innovation?**  *Prompts:*  *-Have you had any user feedback or carried out any evaluation of the patient’s view of the innovation?*  *- Does the innovation require substantial input from the patient or their immediate carer?*  *-Has the innovation improved accessibility?*  **3. What do front-line staff think of the innovation?**  *Prompts:*   - *Do staff question the value proposition for the innovation (e.g., do they feel that adopting it would jeopardise the quality or safety of patient care, or do they think it is more time-consuming than existing practice)?* - *Would the innovation require staff to do their jobs differently and perhaps take on a new, unwanted, role and identity (e.g. 'data entry person')?* - *Do individuals or teams have the resources, time, space, or support and training to deliver the innovation?* - *Are staff confident to be creative and flexible when implementing the innovation and is there organisational support for this adaptive approach?*   ***4.* Is there anything written up about the evaluation of the innovation?**  *Prompts:*  *-Where?*  *-How broadly has it been disseminated? Who is it disseminated to?*  *- Have you disseminated to service users?*  **5. What was your experience of evaluation?**  *Prompt:*  *-To what extent did you find it valuable?*  *-did the evaluation change the design or implementation of the intervention?*  **6. What were the barriers/facilitators, and key challenges/opportunities?**  *Prompts:*  *-Are there any staffing or funding issues?* |
| Implementation, Spread and Sustainability | **1. Tell me about implementation of the innovation.**  *Prompts:*  *- Do you think it has been implemented successfully, where and by whom?*  *- To what extent has it been embedded into routine practice?*  *- How/ where does the innovation fit into the care pathway and how flexible is it?*  -*What was your experience of implementation?*  **2.** **What are the next steps or plans for the innovation in the future?**  **3. To what extent does the** **innovation need adaptation or further development?**  **4. What are your thoughts on scale up (or roll out) of the innovation?**  **5. What are your thoughts on further evaluation of the innovation?**  **-***Have service users been involved in the evaluation?*  **6. How do you think the condition and the population it affects might change over the next 3-5 years?**  *Prompts:*  *- Is the prevalence likely to increase or decrease?*  *-* *May service users’ needs change e.g., if there was another pandemic*  *- How sustainable do you think this model would be in your organisation and within your practice?*  *- Is there a political or economic context that might influence the roll-out if implemented? E.g., change in prioritisation*  *- Do you have any thoughts on how to sustain the model of care over time?*  **7. How has Covid impacted on implementation of the innovation?**  *- Have you had any feedback from patients?*  **8. To what extent do you think the organisation(s) are likely to have significant restructurings or changes in leadership, mission, or strategy over the next 3-5 years in a way that will impact on this innovation?** |
| Thank you for your time. Have you any questions you want to ask? | |
